# The global variability of diatomaceous earth toxicity: a physicochemical and in vitro investigation

**DOI:** 10.1186/s12995-015-0064-7

**Published:** 2015-07-10

**Authors:** C. Nattrass, C. J. Horwell, D. E. Damby, A. Kermanizadeh, D. M. Brown, V. Stone

**Affiliations:** Institute of Hazard, Risk & Resilience, Department of Earth Sciences, Durham University, Durham, DH1 3LE UK; Department of Earth and Environmental Sciences, Ludwig-Maximilians-Universität München, Munich, 80333 Germany; School of Life Sciences, Heriot-Watt University, Edinburgh, EH14 4AS UK; Department of Public Health, Section of Occupational and Environmental Health, University of Copenhagen, Copenhagen, DK-1014 Denmark

**Keywords:** Diatomaceous earth, Cristobalite, Crystalline silica, Cytotoxicity, Haemolysis, Variability

## Abstract

**Background:**

Diatomaceous earth (DE) is mined globally and is potentially of occupational respiratory health concern due to the high crystalline silica content in processed material. DE toxicity, in terms of variability related to global source and processing technique, is poorly understood. This study addresses this variability using physicochemical characterisation and in vitro toxicology assays.

**Methods:**

Nineteen DE samples sourced from around the world, comprising unprocessed, calcined and flux-calcined DE, were analysed for chemical and mineral composition, particle size and morphology, and surface area. The potential toxicity of DE was assessed by its haemolytic capacity, and its ability to induce cytotoxicity or cytokine release by J774 macrophages.

**Results:**

The potential toxicity of DE varied with source and processing technique, ranging from non-reactive to as cytotoxic and haemolytic as DQ12. Crystalline silica-rich, flux-calcined samples were all unreactive, regardless of source. The potential toxicity of unprocessed and calcined samples was variable, and did not correlate with crystalline silica content. Calcium-rich phases, iron content, amorphous material, particle size and morphology all appeared to play a role in sample reactivity. An increased surface area was linked to an increased reactivity in vitro for some sample types.

**Conclusions:**

Overall, no single property of DE could be linked to its potential toxicity, but crystalline silica content was not a dominant factor. Occlusion of the potentially toxic crystalline silica surface by an amorphous matrix or other minerals and impurities in the crystal structure are suggested to pacify toxicity in these samples. In vivo verification is required, but these data suggest that crystalline silica content alone is not a sufficient indicator of the potential DE hazard.

**Electronic supplementary material:**

The online version of this article (doi:10.1186/s12995-015-0064-7) contains supplementary material, which is available to authorized users.

## Introduction

Diatomaceous earth (DE), a sedimentary deposit of silica-rich diatom frustules, is mined globally for a range of purposes, including its commercial value in the filler and filter aid industries [1, 2]. Diatomite deposits are mainly composed of amorphous silica or opal diatom skeletons – the dominant diatom species of which can vary among deposits [2] – and can be interspersed with contaminant minerals, such as clays or carbonates [1]. Extracted material is processed by a series of grinding, calcination and classification techniques to give a wide range of end products. During this process, the diatomite deposit (unprocessed DE) is treated at ~1000 °C with (flux-calcined) or without (calcined) the presence of a fluxing agent, usually sodium carbonate [1, 3]. During calcination, the amorphous silica is converted to crystalline silica, predominantly in the form of cristobalite [2, 3]. The potential for crystalline silica to cause silicosis is well established [4–6], and quartz and cristobalite are classified as Group 1 carcinogens [7, 8]; therefore, exposure to processed DE has the potential to cause chronic respiratory disease.

A number of epidemiology studies assessing the respiratory DE hazard show increased mortality [9, 10], pneumoconiosis [11–14], increased risk of lung cancer [9, 10, 15] and other lung diseases [16] in DE workers, compared to unexposed populations. These findings are supported by radiographic evidence, which shows a strong relationship between exposure and the risk of opacities in chest X-rays indicative of silicosis [17]. However, these studies only focused on DE deposits from California, USA, and little is known about how the physicochemical characteristics of DE vary globally. As such, these data may not inform the health hazard of DE worldwide; the few studies at other locations are much less detailed. The largest of these studies, in Iceland, showed a non-significant increase in lung cancer for DE workers [18]. Beskow [19] and Ebina et al. [20] found signs of silicosis in DE workers in Sweden and Japan, but these were based on a small number of cases; whereas, Joma et al. [21] found no signs of pneumoconiosis in DE-exposed workers in the Netherlands, but airflow in the lung was reduced after exposure.

Some of the above studies include evidence of silicosis-type pathology [12, 19, 20, 22], or strong, positive correlations between crystalline silica content and level of observed disease [9, 17, 23]. However, other clinical and epidemiological studies have demonstrated no link between crystalline silica exposure and the pathogenicity observed in DE workers [14], and showed pathological changes in the lungs which differ from typical quartz-induced silicosis [22]. Generally, crystalline silica content is shown to be very low in unprocessed samples (0–4 wt.%), high in calcined samples (>10 wt.%), and highest in flux-calcined samples (>20 wt.%) (e.g. [9, 24]). It is often difficult to distinguish among these different types of DE exposures in epidemiological studies.

Toxicology studies allow differences between processed and unprocessed DE to be studied systematically. As with epidemiological studies, there is discrepancy among toxicological studies as to whether crystalline silica content is the determinant factor for disease. This knowledge is essential for the effective risk management of worker safety.

Many in vitro studies indicate that the cytotoxicity of DE does not correlate with crystalline silica content, as unprocessed and calcined DE exert a greater cytotoxic effect than cristobalite-rich flux-calcined DE in a number of in vitro assays on different cell lines [24–27]. However, work by Elias and co-workers has shown that cristobalite-rich DE samples (both flux-calcined and those calcined at high temperature) have increased pro-carcinogenic potential in Syrian hamster embryos compared to unprocessed DE and DE calcined at lower temperature [26, 28]. Exposure to both unprocessed and flux-calcined DE also resulted in an increase in abnormal nuclei formation (a marker of genotoxicity) in Chinese hamster ovary cells, with the damage more significant than quartz and cristobalite standards [24].

In vivo studies of unprocessed, amorphous DE show it has the potential to be pathogenic, causing acute/sub-acute inflammation in rats 60 days after a single intratracheal injection of 10 mg DE, an effect that decreased with time [29]. While inhalation of unprocessed DE (170 million particles per cubic foot (mppcf)) by guinea pigs for 39–44 h/week for 24 months resulted in fibrosis, calcined, cristobalite-rich DE was seen to cause more severe fibrosis more rapidly [30]. However, in a separate study, exposure via inhalation to 5–50 mppcf flux-calcined DE, consisting of 61 % cristobalite, for 30 h/week up to 30 months caused no body weight loss or pulmonary fibrosis in rats, guinea pigs or dogs [31].

Previous studies have focussed on few samples and single locations and, therefore, have not been able to investigate the source-dependent compositional and morphological variability of DE and the effect of this variability on toxicity endpoints. This study overcomes this limitation by determining the potential toxicity of DE sourced from seven quarries across the world, and investigating the physicochemical characteristics of the material to understand the properties that affect DE toxicity. Samples chosen cover a spectrum of deposit types, purities and processing techniques. Physicochemical analyses assessed sample composition, morphology, particle size and surface area. Haemolysis allowed for the assessment of particle-induced membrane damage, while cytotoxicity and cytokine release from macrophages were used to assess potential particle toxicity and the potential to induce inflammation.

## Materials and methods

Nineteen DE samples were sourced from mines around the world to account for the global variability of DE deposits (Table 1). A range of DE products that encompass the spectrum of compositional characteristics and processing techniques available were selected using geochemical data obtained though the European Industrial Minerals Association (IMA-Europe). The sample set comprised unprocessed, calcined and flux-calcined samples, and included filler and filter aid grades of DE, which are determined by post-calcination size classification. Samples were chosen from a range of source deposits, including those with impurities (carbonates and clays). Unprocessed samples, and the equivalent sample post-processing, were sourced where possible in order to directly establish the effects of processing.

**Table 1:** Information for diatomaceous earth samples, including: particle size distribution, surface area and chemical composition

Sample information				Particle size distribution (c.v. %)		BET surface area (m^2^/g)		Chemical composition (wt.%)							
Sample ID	Source	Process	Grade	<4 μm	<10 μm	Mean	s.d.	SiO_2_	TiO_2_	Al_2_O_3_	Fe_2_O_3_	MgO	CaO	Na_2_O	K_2_O
DE_05^#a^	Spain	U	Filler	11.3	38.8	7.5	0.7	90.47	0.05	0.91	0.38	0.34	6.96	0.65	0.14
DE_11^b^	Spain	U	Filler	12.4	47.3	6.3	0.4	87.26	0.05	1.40	0.42	0.49	8.96	1.04	0.17
DE_13^c^	France	U	Filter aid	7.8	28.0	22.9	0.2	89.02	0.64	4.42	3.22	0.36	0.66	0.38	0.35
DE_15^d^	France	U	Filter aid	7.9	28.5	23.8	0.3	87.40	0.70	4.73	3.99	0.49	0.74	0.19	0.34
DE_16^d^	France	C	Filter aid	6.7	25.0	3.8	0.1	88.03	0.66	4.50	3.86	0.39	0.66	0.17	0.33
DE_18	China	C	Filter aid	7.3	28.1	3.1	0.1	93.49	0.15	3.71	1.67	0.35	0.08	−0.04	0.50
DE_20^#^	Mexico	C	Filter aid	6.8	25.3	4.0	0.1	92.00	0.24	5.07	1.95	0.34	0.30	−0.13	0.20
DE_21	USA-1^e^	C	Filter aid	6.2	21.7	5.7	0.2	87.83	0.32	6.57	1.81	1.02	0.54	0.51	1.02
DE_22	USA-1	C	Filler	25.4	71.5	10.6	0.2	93.24	0.17	3.82	1.15	0.65	0.17	0.03	0.59
DE_23	USA-1	C	Filter aid	21.8	65.5	6.8	0.2	93.88	0.15	3.03	1.05	0.69	0.30	0.21	0.47
DE_24^#^	Mexico	C	Filter aid	12.1	45.0	5.9	0.2	93.63	0.19	3.89	1.49	0.33	0.22	0.01	0.18
DE_06^a^	Spain	FC	Filler	8.5	34.4	1.1	0.1	91.27	0.03	0.67	0.35	0.27	6.25	1.00	0.09
DE_07	USA-1	FC	Filler	10.6	31.6	1.3	0.0	91.67	0.15	3.00	1.07	0.53	0.29	2.69	0.48
DE_08^#^	USA-2^e^	FC	Filler	9.2	28.7	1.3	0.1	93.07	0.06	1.12	1.99	0.51	0.30	2.89	0.03
DE_09	Chile	FC	Filler	9.8	33.4	1.5	0.1	93.04	0.10	1.82	0.95	0.23	1.29	2.15	0.34
DE_10^#^	Mexico	FC	Filler	8.7	35.3	1.7	0.1	93.64	0.10	2.05	0.80	0.14	0.32	2.59	0.12
DE_12^b^	Spain	FC	Filler	8.5	33.4	1.0	0.1	90.67	0.03	1.12	0.35	0.31	6.64	0.69	0.10
DE_14^c^	France	FC	Filter aid	6.4	17.0	1.1	0.1	87.36	0.58	4.10	2.61	0.40	0.62	3.17	0.39
DE_17	China	FC	Filter aid	4.6	11.4	1.0	0.0	91.68	0.13	3.12	1.44	0.27	0.11	2.22	0.51

Table sorted by process: *U* unprocessed, *C* calcined, *FC* flux-calcined, *c.v.* cumulative volume, *s.d.* standard deviation (*n* = 3). ^#^The fine fractions of these samples were separated for use in toxicology assays. ^e^ Samples were sourced from two separate deposits in the USA and so are denoted as USA-1 and USA-2.^a–d^ Unprocessed samples and their corresponding processed samples

### Separation of the fine fraction

Fine fractions (close to PM10) were separated from 5 bulk samples for toxicological assessment alongside their bulk counterparts (Table 1). The samples, which comprise a range of crystalline silica contents, bulk impurities, and processing techniques, were separated by dry-resuspension as previously described by Moreno et al. [32]. Briefly, the sample was suspended in a horizontal rotating drum with a baffle (1.5 rpm). Airflow of 6 l/min was passed through this system, which carried suspended particles through a gravitational settling chamber, where coarser particles were deposited, and the fine fraction continued through a Negretti elutriation filter system and was collected on a polycarbonate filter. The particle size distribution of the separated fine fractions was determined by SEM image analysis.

### Physicochemical characterisation

Physiochemical analyses were carried out on bulk samples (because of the mass required by some techniques). The crystalline silica polymorph and relative crystalline silica contents were measured by X-ray diffraction position sensitive detection (XRD-PSD; Bruker D8 Advance, Durham University) from 5 to 90° 2θ. Samples were ground to a fine powder and compacted into a well using the knife-edge of a spatula to ensure random crystal orientation [33]. The intensity of the primary peak (26.6 °2θ for quartz and 21.8 °2θ for cristobalite) was used as a proxy for the relative amounts of crystalline silica. To account for the different diffraction intensities of quartz and cristobalite, the peak heights of pure-phase quartz and cristobalite standards, run under the same conditions, were used to normalise the relative peak heights in the samples. This allowed the addition of peak intensities of quartz and cristobalite to give relative total crystalline silica contents amongst the samples. Bulk chemical compositions were measured by X-ray fluorescence (XRF; PANalytical Axios Advanced X-ray fluorescence spectrometer, University of Leicester). Particle sections were produced in polished resin blocks, coated with 25 nm carbon, and imaging and elemental analysis were performed by scanning electron microscopy (SEM) and energy dispersive X-ray spectroscopy (EDS) at 15 kV (Hitachi SU-70 FEG SEM, Durham University). The elemental composition of cristobalite in individual particles, and amorphous material in cristobalite-containing particles was measured in samples where these particles were clearly seen.

Particle size distributions were analysed by laser diffraction using a Coulter LS analyser (Durham University), with polarization intensity differentiation scattering (PIDS) to analyse particles in the range of 0.04–2000 μm diameter (all samples fell within this range). Data are presented as cumulative volume (c.v.) % and are an average of two 90 s measurements, analysed by Fraunhofer theory.

Qualitative analysis of dominant diatom morphology was performed on all samples by mounting particles on polycarbonate discs, which were adhered to aluminium stubs by carbon pads. These were coated with 25 nm gold/palladium and imaged at 8 kV by SEM. Quantitative assessment (1000–2000 particles per sample) of particle size and the abundance of fibre-like particles (defined by an aspect ratio >3 [34]), was conducted on the five fine fractions used in toxicology experiments and their corresponding bulk samples (DE_05, DE_08, DE_10, DE_20 and DE_24).

Surface area was analysed by nitrogen adsorption measurements at 77 K using a TriStar 3000 instrument (Durham University). Samples were dried in nitrogen gas at 120 °C overnight. The Brunauer-Emmett-Teller (BET) theory was applied to measurements at relative pressures of 0.05–0.24, and the results are the mean of three repeated measurements.

### In vitro toxicology

Haemolysis is a measure of the ability of particles to rupture cell membranes, and has been shown to be a good indicator of the pro-inflammatory potential of crystalline silica [35]. Haemolysis was performed on all 19 bulk samples and 5 separated fine fractions by treating red blood cells with 63–1000 μg/ml DE powder for 1 h. A volume of 1 ml of sheep blood in Alsever’s solutions (Oxoid Ltd.) was centrifuged at 5000 rpm for 2 min and the supernatant removed. Isolated red blood cells were washed three times with saline and 100 μl of cells were added to 3.6 ml saline. Particle suspensions of 1 mg/ml particles in saline were sonicated for 20 min, serially diluted to final concentrations, and 150 μl of the particle suspensions added to 96 well plates in triplicate. Next, 75 μl of the prepared blood was added to each well, the plate covered and placed on an orbital shaker for 1 h. Post-exposure, the plate was centrifuged at 250 rcf for 5 min, 100 μl of the supernatant transferred to a new plate and absorbance measured at 540 nm (SpectraMax M5, Heriot-Watt University).

Cytotoxicity was measured using alamarBlue® (a measure of mitochondrial enzyme activity) and lactate dehydrogenase (LDH; a measure of membrane integrity) assays (Heriot-Watt University). These assays were performed on the five fine fractions, their corresponding bulk samples, two bulk samples chosen due to their haemolytic potential (DE_11 and DE_22), as well as a further unprocessed bulk sample (DE_15). The samples were suspended in RPMI medium containing 10 μl/ml L-glutamine, 10 μl/ml penicillin and streptomycin, and 10 % bovine foetal serum (complete medium), and sonicated for 20 min. J774 macrophages were seeded in a 96 well plate (5 × 10^4^ cells per well) and exposed to 100 μl of DE suspension in concentrations of 8, 16, 31, 63, 125, 250, and 500 μg/ml for 24 h at 37 °C and 5 % CO_2_. Following exposure, the supernatant was removed and stored at −80 °C for LDH and cytokine analysis. A solution of 1 mg/ml alamarBlue® reagent (resazurin sodium salt; Sigma) in saline was diluted 1:10 in complete medium and 100 μl added to the cells. The plate was incubated for 4 h and fluorescence measured at excitation at 560 nm and emission at 590 nm. LDH release from macrophages was measured by adding 10 μl of cell supernatant to 50 μl 1 mg/ml NADH in 0.75 mM sodium pyruvate, incubating for 30 min at 37 °C in 5 % CO_2_, adding 50 μl of 2 mg/ml 2,4-dinitrophenylhydrazine in 1 M HCl and incubating for 20 min at room temperature in the dark, before adding 50 μl 4 M sodium hydroxide and measuring the absorbance at 550 nm.

Cytokine production was measured as a marker of inflammation using BD™ Cytometric Bead Array cytokine flex sets (bead based immunoassay; BD Biosciences, Heriot-Watt University). Flow cytometry was used to discriminate between different bead populations based on size and fluorescence, according to the manufacturer’s instructions. Keratinocyte chemoattractant (KC), interleukin 1β (IL-1β), tumour necrosis factor alpha (TNF-α) and interleukin 10 (IL-10) were measured for cells treated with 125 μg/ml or lower of the fine fractions of DE only. TNF-α and IL-1β are pro-inflammatory cytokines associated with silica induced toxicity [36, 37], IL-10 is an anti-inflammatory cytokine providing information on the balance between pro- and anti-inflammatory signalling, and KC induces neutrophil and macrophage chemotaxis. For samples demonstrating cytotoxicity, the LC20 concentration, two times the LC20 and half the LC20 would generally be assessed to investigate cytokine production. However, in low toxicity samples, where an LC20 was not reached, only a concentration of 125 μg/ml was chosen to evaluate the inflammatory response.

Cells treated with the five fine fractions were imaged by light microscopy. J774 cells (2.5 × 10^5^) were treated with 600 μl of 63 μg/ml DE in a 24 well plate. Cells were scraped from the plate and 50 μl of the cell suspension diluted in 300 μl saline and centrifuged onto a microscope slide at 1160 rcf for 5 min. The treated cells were dried and stained with Diff-Quik (Fisher Scientific). Briefly, slides were dipped in methanol, then Eosin G in phosphate buffer and Thiazine dye in phosphate buffer, rinsed with H_2_O and air dried.

In all assays, Triton-X was used as a positive control and untreated cells as a negative control. DQ12 was used as a positive crystalline silica standard and TiO_2_ as a negative particle standard. A calcite standard was also used in the alamarBlue® and haemolysis assays, due to a high calcite content in some samples. Results are presented as the relative percentages of the positive and negative controls.

### Statistical analysis

Student’s *t*-test and ANOVA general linear model with a Tukey’s post-hoc test were performed to determine the significance of differences among samples in the in vitro assays (Minitab 15). Pearson’s correlation test was used to determine significant correlations amongst different physicochemical characteristics and toxicological results (* *p* <0.05, ** *p* <0.01, *** *p* <0.001).

## Results

### Physicochemical characterisation

#### Bulk chemical composition

The bulk chemical composition was variable across deposit sources and processing technique (Table 1). All samples were predominantly SiO_2_, which ranged from 87 to 94 wt.%. Samples contained up to 7 and 4 wt.% Al_2_O_3_ and Fe_2_O_3_, respectively. Fe_2_O_3_ content was highest in French samples and Al_2_O_3_ contents were lower in Spanish samples compared to other locations. Samples from Spain had CaO contents up to 9 wt.%, substantially higher than from all other locations. On average, flux-calcined samples contained significantly more Na_2_O than unprocessed and calcined samples from all locations (*p* <0.001), and contained less Al_2_O_3_ than calcined samples. Other elements comprised <1.3 wt.% in all samples.

#### Crystalline silica

**Fig. 1:**
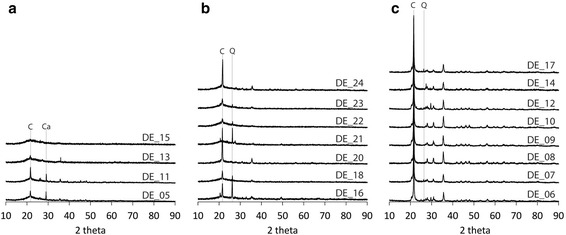
XRD patterns of (**a**) unprocessed, (**b**) calcined and (**c**) flux-calcined DE. *C* cristobalite, *Q* quartz, *Ca* calcite. Only primary peaks are labelled for clarity. Quartz was not detected in unprocessed samples by XRD

Crystalline silica content varied amongst samples, but was the dominant mineral phase in flux-calcined and calcined samples (Fig. 1). Flux-calcined samples were predominantly cristobalite; though, samples from China and USA also contained small quantities of quartz (Fig. 1c). Conversely, calcined samples contained cristobalite as well as quartz, with the exception of cristobalite-only Mexican samples DE_20 and DE_24 (Fig. 1b). Quartz was the dominant crystalline silica polymorph in calcined samples DE_16 and DE_21, whereas cristobalite was the dominant phase in the other calcined samples. On average, cristobalite peak intensity was three times higher in flux-calcined samples than in calcined samples, as calcined samples retained more amorphous material and a proportion of the crystalline silica was quartz. These differences in abundance were substantiated by qualitative SEM imaging. Unprocessed samples were predominantly amorphous and only contained small amounts of cristobalite (Fig. 1a) or, rarely, quartz was observed in all four samples.

**Fig. 2:**
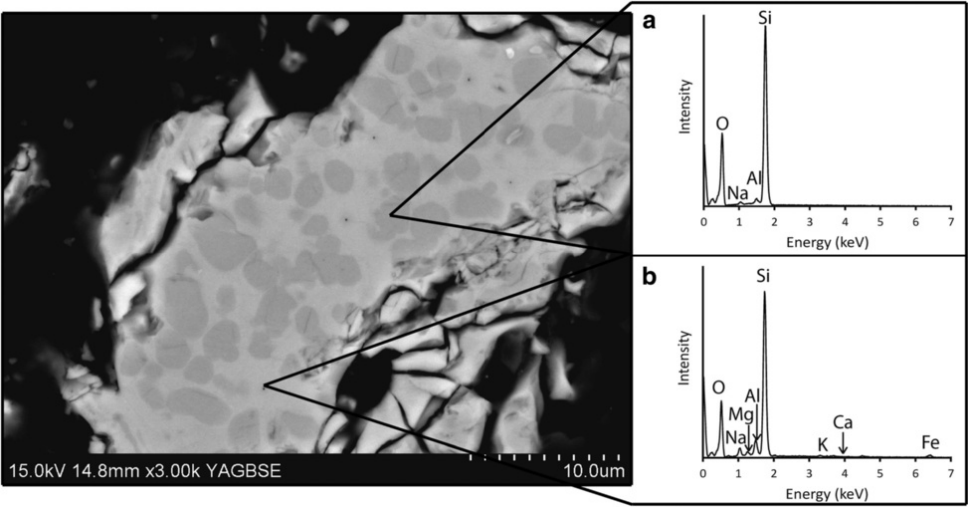
Backscatter image and EDS spectra of flux-calcined DE particle. Backscatter image of DE particle in a flux-calcined sample with cristobalite patches (*dark grey*; identified by cracking and cristobalite is only crystalline phase in this sample by XRD) surrounded by an amorphous matrix (*light grey*). EDS spectra of (**a**) cristobalite patches showing Al and Na impurities and (**b**) amorphous matrix containing Na, Mg, Al, Ca, K and Fe impurities. These are representative spectra from ~800 spot analyses of cristobalite patches and amorphous matrices (400 of each). Some cristobalite patches also contained Ca and Fe, and some amorphous material also contained Ti and P

By backscatter SEM, crystalline silica appeared as dark grey patches within lighter grey matrices of amorphous material (Fig. 2). In samples that contained both quartz and cristobalite, cristobalite could often be identified by characteristic ‘fish-scale’ cracking, indicative of the particle having undergone the transition from the high-temperature beta form to the low-temperature alpha form [38]. By EDS analysis on cristobalite-only samples and ‘fish-scale’ patches in samples containing both polymorphs, Na, Al, Fe and Ca were detected in the cristobalite (Fig. 2). The amorphous matrix was enriched in impurities compared to the cristobalite, containing Na, Al, Fe, Ca, Mg, K, Ti and P (Fig. 2).

#### Contaminant phases

**Fig. 3:**
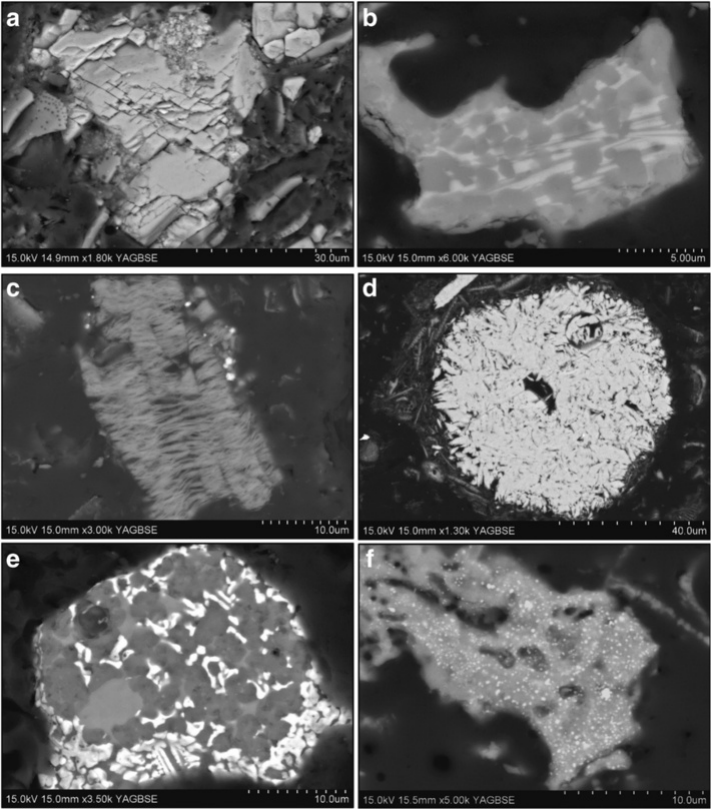
Backscatter images of contaminant phases in DE. **a** a pure calcite particle in unprocessed DE from Spain (DE_05), **b** a particle containing calcium silicate (*very light grey*) and cristobalite patches (*dark grey*) in flux-calcined DE from Spain (DE_06), **c** a clay mineral particle in an unprocessed sample from France (DE_13), **d** a large iron phosphate particle in unprocessed DE from France (DE_13), and **e**-**f** iron-rich patches within particles in French (DE_16) and Mexican (DE_24) calcined samples

Spanish samples contained calcite (Figs. 1 and 3a); this was most prominent in the unprocessed samples (DE_05, DE_11), with only traces remaining in the corresponding flux-calcined samples (DE_06, DE_12). Calcium silicates were observed in abundance in all Spanish samples (Fig. 3b).

Clays were observed by SEM-EDS (texturally, and by their high Al contents) and were most prevalent in French samples (e.g. Fig. 3c), but were also observed in samples from USA-1, Mexico and China. Distinct clay phases were not identifiable using textural features or chemical composition, and quantities were too low for detection by XRD.

In the French, unprocessed samples, iron-rich particles of up to 70 μm were observed and these were iron and titanium-rich or iron and phosphorus-rich particles (Fig. 3d). These larger particles were not observed in the calcined and flux-calcined samples from France; instead, small iron-rich patches were seen throughout particles and sometimes in cracks and pore spaces (Fig. 3e). Some of these deposits were iron and titanium-rich but were mostly iron and silicon. In calcined samples where the corresponding unprocessed samples were not available, iron-rich patches were seen throughout particles (Fig. 3f).

#### Physical properties

Bulk samples contained between 11 and 71 c.v. % particles <10 μm and between 5 and 25 c.v. % particles <4 μm in diameter (Table 1), analogous to the thoracic and respirable fractions, respectively [39]. Flux-calcined samples were coarser than unprocessed and calcined samples for both filler and filter aid grade samples. Calcined samples had a wide range of particle size distributions, containing between 22 and 71 c.v. % <10 μm material (Table 1). The maximum particle diameter observed in the separated fine fractions was 51 μm, reduced from 73 μm in the bulk fractions. Additionally, 83–99 % of particles, by number, in the fine fractions were <10 μm in diameter, compared to 73–96 % in the bulk samples (Table 2). The separated fraction of flux-calcined samples was coarser (median diameter 1.5–2.6 μm) than the fine fractions of calcined and unprocessed samples (median diameters 0.8 μm; Table 2). These data are not comparable to laser diffraction data, which present equivalent spherical diameter in volume % so are heavily mass-biased.

**Table 2:** Particle size measured by SEM image analysis

Sample	Maximum particle diameter (μm)		Median particle diameter (μm)		Particles <10 μm diameter (number %)		Fibre-like particles (%)	
	Bulk	Fine	Bulk	Fine	Bulk	Fine	Bulk	Fine
DE_05	26.9	18.5	1.7	0.8	96.6	99.2	17.9	15.4
DE_20	52.0	31.5	1.9	0.8	94.1	96.5	21.2	18.8
DE_24	47.0	17.3	1.8	0.8	96.5	99.0	17.8	16.4
DE_08	73.0	43.7	5.0	1.5	73.2	82.7	3.3	5.7
DE_10	46.2	51.3	4.1	2.6	77.9	83.0	4.9	9.8

Includes maximum particle diameter, median particle diameter, the number % of particles <10 μm, and the number % of fibre-like particles in five bulk samples and their corresponding fine fractions

**Fig. 4:**
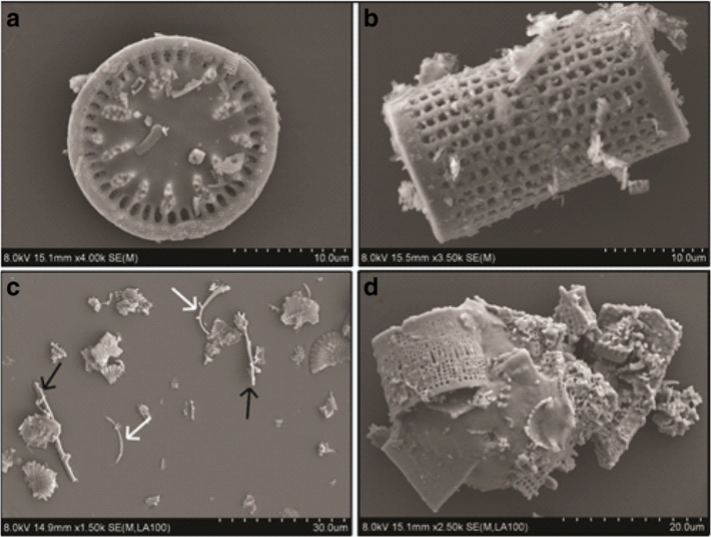
SEM images of examples of DE morphologies. **a** disc-shaped DE found in Chilean, Chinese, Mexican and Spanish samples, **b** cylindrical diatoms found in French and USA-2 samples, **c** fibre-like rods (*black arrows*) and diatom rinds (*white arrows*), and **d** a large agglomeration of fused diatoms in a flux-calcined DE sample

Particle morphology varied substantially amongst samples as the dominant diatom species qualitatively varied with sample source. Samples from Chile, China, Mexico and Spain contained predominantly disc-shaped diatoms, whereas samples from USA-2 and France consisted mainly of cylindrical diatoms (Fig. 4). Samples sourced from USA-1 contained a number of different diatom species and morphologies, including disc-shaped, and long, thin diatoms (Fig. 4c). Most particles were fragments of diatoms and, therefore, particle morphology varied substantially within individual samples. Processed samples contained some sintered particles and fused pores, and this was most evident in flux-calcined samples, where large agglomerates of diatoms were observed (Fig. 4d).

In the five bulk samples and equivalent fine fractions, between 3 and 21 % of particles, by number, were classified as fibre-like (Table 2). The total number of fibre-like particles was 2 to 6 times lower in flux-calcined samples than in unprocessed or calcined samples, for both bulk samples and fine fractions (Table 2).

Surface area did not vary much among flux-calcined samples and was always <1.7 m^2^/g. However, in calcined and unprocessed samples, surface area varied substantially, ranging from 3.1 to 10.6 m^2^/g in calcined samples and 6.3 to 23.8 m^2^/g in unprocessed samples (Table 1).

### In vitro toxicology

#### Haemolysis

**Fig. 5:**
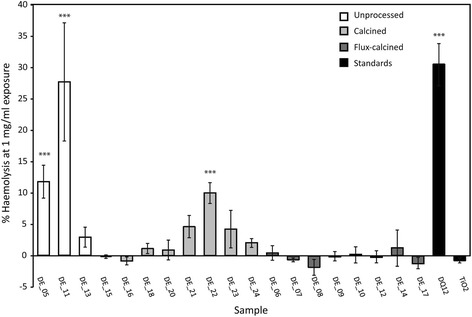
Haemolytic potential of DE samples. Percent haemolysis relative to an untreated control, of sheep red blood cells post-exposure to 1 mg/ml of unprocessed (*white*), calcined (*light grey*) and flux-calcined (*dark grey*) DE and positive (DQ12) and negative (TiO_2_) standards (*black*). *Error bars* represent standard error (*n* = 3), *** *p* = <0.001 difference from untreated control

Haemolysis results are shown in Fig. 5 for bulk samples. Haemolysis caused by the fine fractions did not differ significantly from their corresponding bulk samples and, hence, are not shown. Unprocessed samples from Spain (DE_05 and DE_11) were two of the most haemolytic samples, with DE_11 as haemolytic as DQ12. The most haemolytic calcined sample, DE_22, was one third as haemolytic as DQ12 at the highest concentration. All other samples were not significantly haemolytic at any concentration tested, including the calcite standard (data not shown).

#### Cytotoxicity

**Fig. 6:**
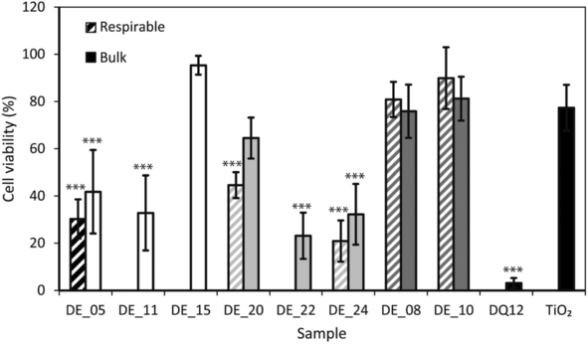
Cell viability of J774 macrophages exposed to DE. Cell viability relative to an untreated control (% of untreated control–positive control), measured by the alamarBlue® assay, of J774 macrophages exposed to 500 μg/ml of bulk (*solid*) or the fine fraction (*hashed*) of unprocessed (*white*), calcined (*light grey*) and flux-calcined (*dark grey*) DE, and positive (DQ12) and negative (TiO_2_) standards (*black*) for 24 h. No data for fine fractions of DE_11, DE_15 and DE_22. *Error bars* represent standard error (*n* = 4), *** *p* = <0.001 difference from untreated control

In the alamarBlue® assay (Fig. 6), there was no statistically significant difference in cytotoxicity of bulk DE samples and their separated fine fractions. Bulk flux-calcined samples, and DE_20 and DE_15, TiO_2_ and calcite did not differ from the untreated control. All other samples exhibited a cytotoxic response and the cytotoxicity of DE_24 (bulk and fine), DE_22, DE_11 and the fine fraction of DE_05 did not differ significantly from DQ12 (*p* > 0.2). LDH release data were in broad agreement with cell viability measurements via the alamarBlue® assay, in that flux-calcined samples were less cytotoxic than unprocessed and calcined samples (Additional file 1).

#### Cytokine release

**Fig. 7:**
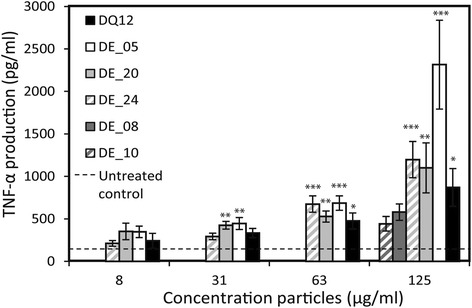
TNF-α production by macrophages exposed to the fine fractions of DE. Exposure to unprocessed (*white*), calcined (*light grey*) and flux-calcined (*dark grey*) DE and a positive standard (DQ12) for 24 h. *Error bars* represent standard error (*n* = 8). * *p* = <0.05, ** *p* = <0.01, *** *p* = <0.001 compared to untreated control

Only TNF-α was produced by macrophages in concentrations greater than untreated cells following exposure to the fine-fraction samples (Fig. 7, data for other cytokines given in Additional file 1). DE_05, DE_20 and DE_24 all increased TNF-α production, with DE_05 inducing the greatest TNF-α production at 125 μg/ml, which was 2.7 times greater than that released following treatment with DQ12. For the calcined samples (DE_20, DE_24), the induction of TNF-α production by treated cells was comparable to that induced by DQ12. In contrast, DE_10 and DE_08 did not enhance TNF-α production.

#### Cell imaging

**Fig. 8:**
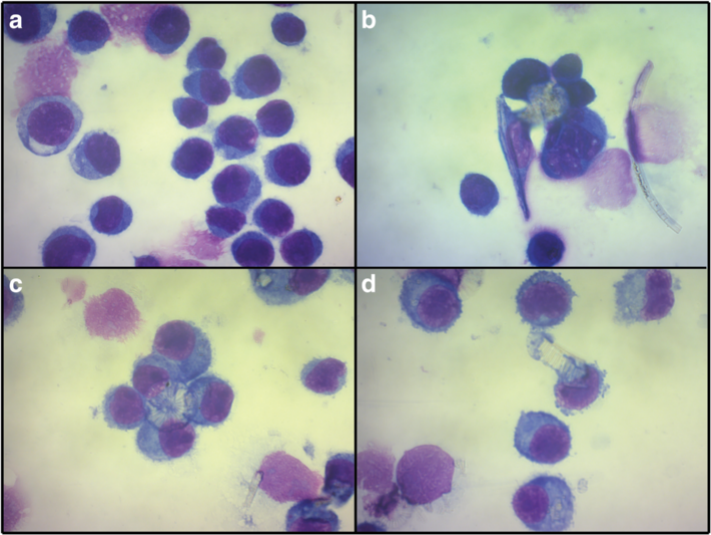
Frustrated phagocytosis in macrophages treated with DE. Light microscopy image of (**a**) untreated macrophages, and (**b**-**d**) macrophages treated with DE showing indicators of frustrated phagocytosis

Imaged cells treated with the fine fractions of DE and an untreated control are shown in Fig. 8. In the treated cells, there was some degree of frustrated phagocytosis, with instances of numerous cells attempting to phagocytose the same particle and particles not fully engulfed (Fig. 8b-d). Qualitatively, evidence of frustrated phagocytosis was more abundant in cells treated with calcined or unprocessed samples, compared to flux-calcined samples, but were observed in all cases.

## Discussion

### The variability of DE properties

DE deposits vary in composition and diatom species providing DE particles with a range of physical and chemical properties. Treatment of DE by calcination is used to optimise these properties for use in the filtration and filler industries, during which amorphous silica crystallises [1, 2]. Here, crystalline silica contents and polymorphs varied substantially amongst DE samples from different locations, with processing playing a key role. Flux-calcined samples were the most crystalline silica-rich, and cristobalite was the dominant crystalline silica polymorph. Most calcined samples contained both cristobalite and quartz and there is some evidence that the quartz formed during calcination (based on data from the unprocessed DE_15 and calcined DE_16 samples). This distribution of crystalline silica polymorphs by processing technique has also been observed by Ghiazza et al. [27] and Elias et al. [28]. The preferential crystallisation of cristobalite in flux-calcined samples is likely due the addition of the fluxing agent: doping of amorphous silica with sodium salts promotes cristobalite crystallisation instead of quartz at the temperatures used for calcination [40, 41]. It is unknown why quartz preferentially forms in some calcined samples, whereas in others only cristobalite was observed (Mexican samples DE_20 and DE_24). Unprocessed samples contained trace quantities of cristobalite and quartz; the cristobalite in unprocessed samples likely forms by diagenesis of amorphous silica over time, or is possibly from other sources, such as volcanic deposits [2].

Cristobalite formed in patches throughout particles, crystallising within a matrix of amorphous material (Fig. 2). The cristobalite patches in the samples analysed were impure, containing Al, Na, Ca and Fe (e.g. Fig. 2). Aluminium and Fe ions can substitute for Si in the silica tetrahedra, but require interstitial cations (e.g., Na or Ca) to charge balance the substitution [42], as has been discussed for volcanic cristobalite [43, 44].

Contaminant phases were observed in all samples. Spanish deposits contained calcite (DE_05, DE_11), which would thermally decompose at calcination temperatures (~1000 °C) [45], allowing Ca and Si to form calcium silicates, which were in abundance in flux-calcined samples from Spain. Clay minerals and iron-rich particles are also likely to be broken down during calcination as few individual clay mineral particles or large, iron-rich particles were seen in processed samples compared to unprocessed samples from France. As with Ca in Spanish samples, this frees Fe ions to react with Si, and precipitate in pores and cracks, which was seen in all calcined samples (Fig. 3e-f). The addition of a sodium carbonate flux leads to sodium silicate melt binding with Fe [2], which explains the lack of iron-rich particles in flux-calcined samples.

Physical properties varied substantially among the samples. Flux-calcined particles had complex morphologies, and were coarser and had lower surface area than calcined and unprocessed samples, due to sintering of particles into large agglomerates and the closure of pore spaces [3]. The morphology of diatom frustules varied with source (Fig. 4); however, the abundance of fragmented frustules in all samples meant diatom species, alone, could not be used as a good proxy for particle morphology.

### The variability of the potential toxicity of DE

Previous studies of DE toxicity insufficiently constrain the respiratory hazard posed by exposure to DE deposits worldwide: epidemiology studies have mainly focussed on DE workers in a single location (California) (e.g. [9, 10, 14–17]), and are unable to distinguish between exposure to unprocessed or processed DE, while toxicology studies rarely state the sample source and investigate few samples. Therefore, variability among deposits and processing techniques has not been fully considered. Here, we show that DE is not a single entity, with a toxic potential that ranges from unreactive to as haemolytic or cytotoxic as the positive standard α-quartz (DQ12).

Unprocessed samples from Spain (DE_05 and DE_11) were haemolytic and induced significant cytotoxicity to macrophages. Furthermore, DE_05 induced a large increase in TNF-α release from treated macrophages relative to processed samples, indicating the potential for unprocessed DE to incite inflammation if inhaled. However, unprocessed samples from France did not induce any measurable haemolysis or cytotoxicity, suggesting that the potential toxicity of DE can vary by deposit source.

Flux-calcined samples all displayed low haemolytic potential, cytotoxicity and cytokine release from treated cells. This suggests that short-term exposure to flux-calcined samples may not result in toxicity or an inflammatory response. There was no variation in the reactivity of flux-calcined samples from different locations, suggesting that flux-calcination is key to dampening the adverse effects attributed to DE. This is likely due to alteration of the physicochemical properties of DE during flux-calcination and is discussed in detail below.

DE_22 from USA-1 was the only calcined sample that elicited a haemolytic response and was also one of the most cytotoxic samples analysed. This introduces the possibility that data in epidemiological studies from the USA may not be representative of likely disease outcomes worldwide. However, other calcined samples from USA-1 were non-haemolytic, emphasising the variable reactivity that exists amongst samples from a single deposit source. Calcined samples from Mexico were cytotoxic but not haemolytic. Therefore, as these assays assess different mechanisms of toxicity, and there is a disagreement in sample reactivities among the assays for samples from some locations but not others, different mechanisms may be responsible for the observed reactivity.

### Factors affecting DE toxicity

#### Presence of crystalline silica

**Fig. 9:**
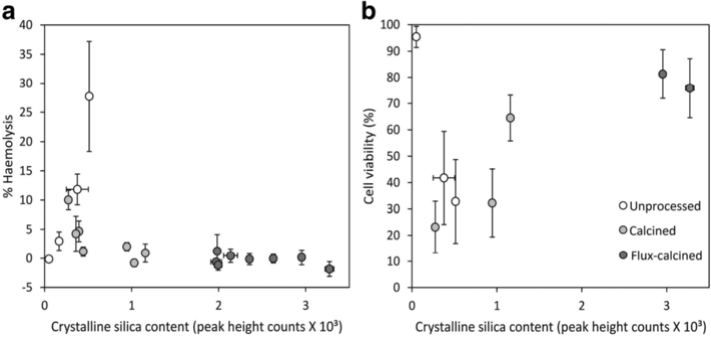
Relationship between crystalline silica content and reactivity in vitro. Correlation of crystalline silica content of DE (measured by the addition of cristobalite and quartz peak heights, accounting for different diffraction power using standards) with (**a**) haemolysis post-exposure to 1 mg/ml bulk DE, and (**b**) cell viability post-exposure to 500 μg/ml bulk DE

Here, total crystalline silica (quartz plus cristobalite) content alone did not control DE reactivity in vitro (Fig. 9), as has also been reported by other in vitro toxicity studies [24–27]. This contrasts with previous epidemiology studies that attribute observed pathologies to crystalline silica in processed DE [e.g. [9, 14]]. Some of these studies are referenced in reviews of crystalline silica toxicity [46, 47], and are used in determining safe exposure limits to crystalline silica [e.g. [48]].

The low reactivity of all flux-calcined samples agrees with previous reports on discrete samples [24, 25, 27, 31]. Ghiazza et al. [27] suggested that the low levels of cytotoxicity, lipid peroxidation and NO synthesis caused by crystalline silica-rich flux-calcined DE was due to the formation of a vitreous rim during flux-calcination, which obscured cell exposure to cristobalite in these samples. No vitreous rim, per se, was observed in this study but the cristobalite is partly occluded, occurring in patches within the otherwise amorphous particles, resulting in a variable percentage of cristobalite at the particle surface. This may account for the lack of correlation between crystalline silica content and potential toxicity. The variable toxicity of crystalline-silica bearing dusts is well established [e.g. [36, 49, 50]], and the hosting of crystalline silica within heterogeneous particles has previously been suggested to explain variations in the toxic potential of quartz-rich coal dusts [49, 51], and to mask the toxicity of cristobalite in volcanic ash [44]. Here, exposure times for the cytotoxicity assays were short (24 h), and the amorphous matrix may have low bio-durability, thereby exposing more cristobalite at the surface of the particle over time. Therefore, future longer exposures and in vivo experiments may be necessary.

Toxicity of the limited surface cristobalite could be further dampened by the presence of cation substitutions (Fig. 2), as structural impurities have been hypothesised to decrease the toxic potential of cristobalite in volcanic ash [44]. Treating quartz with aluminium salts or clay extracts has also been shown to reduce its toxicity and haemolytic potential [52–55], and quartz in coal with clay mineral impurities had lower toxicity than pure, coal-sourced quartz [51]. Therefore, the production of aluminium ions via breakdown of aluminium-rich components of the DE samples could affect the cristobalite exposed at the surface of particles, decreasing the potential toxicity. Conversely, treating quartz with ferric or ferrous iron does not change its toxic potency in vivo [52] and can decrease its haemolytic potential [54]. However, Fubini et al. [56] showed iron may increase silica toxicity via production of free radicals. Therefore, the role of iron in determining DE crystalline silica toxicity remains unclear.

#### Reactivity of contaminant phases

Some samples with little to no crystalline silica were highly reactive, indicating contaminant phases may control the in vitro response in those samples. The presence of calcite could explain the high reactivity of Spanish (calcite-rich) unprocessed samples: Aladdin et al. [57] have shown exposure to calcite alone caused significant cytotoxic effects in human epithelial cells and inflammation in mice, and Diler and Ergene [58] showed that micronuclei and nuclei abnormalities were more common in calcite factory workers than control groups. However, as the calcite standard was non-reactive in both the haemolysis and alamarBlue® assays, it is unlikely that calcite content is solely responsible for the different reactivity of Spanish unprocessed and flux-calcined DE. The presence of clays may also have an effect, as some clays have been shown to be more haemolytic than crystalline silica [59, 60]. However, the lack of clay in processed DE samples and the low reactivity of French, unprocessed samples, suggests that clay minerals are not the cause of DE induced toxicity.

Iron has been hypothesised as a source for free radical generation in DE [26], and was measured in quantities of up to 4 wt.% here and, therefore, could be contributing to the potential toxicity. Bulk iron content did not correlate with either haemolysis or cytotoxicity (Additional file 1); however, further work would be required to determine the co-ordination of iron and its potential to produce free radicals. Calcined samples all contained iron-rich phases but had variable toxicity, suggesting total Fe does not control the reactivity of these samples. Also, previous studies have shown ferric and ferrous oxides to have low cytotoxicity [61], although exposure to ultrafine iron oxide particles has been shown to lead to oxidative stress and a pro-inflammatory response in rats [62, 63]. Soluble iron oxide has also been shown to be cytotoxic to human mesothelioma MSTO-211H cells [64]. However, as soluble components of the DE were non-cytotoxic (see Additional file 1), this is unlikely to be the case here.

Amorphous silica is generally perceived as less toxic than crystalline silica [49] but can be reactive in vitro [65, 66]. The ability of amorphous, unprocessed DE to elicit a haemolytic or cytotoxic response, or induce abnormal nuclei growth in vitro [24, 25, 29], and cause fibrosis in vivo [30], has also previously been demonstrated. Unprocessed samples from Spain, the most haemolytic of all samples, were predominantly amorphous. The cytotoxic (DE_20, DE_22 and DE_24) and haemolytic (DE_22) calcined samples also contained a substantial amount of amorphous material. It is possible that the amorphous phases of these samples are linked to their reactivity, as certain vitreous silicas have been shown to be more haemolytic than quartz [66]. However, the lack of haemolysis caused by French, unprocessed samples, which were also predominantly amorphous, means that amorphous material is unlikely the predominant control on the observed reactivity. The high levels of impurities in processed amorphous material (Fig. 2) may also play a role in its biological reactivity at the surface of the particle, but further work is needed to determine this.

Therefore, although the mineralogical and chemical compositions are likely to play a role in DE toxicity, no definitive link can be made between individual characteristics considered here and the potential toxicity measured in this study.

#### Physical properties

**Fig. 10:**
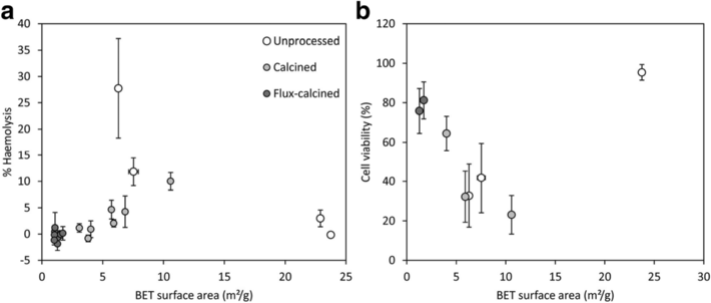
Relationship between BET surface area and DE reactivity in vitro. Correlation between surface area and (**a**) haemolysis post-exposure to 1 mg/ml bulk DE, and (**b**) cell viability post-exposure to 500 μg/ml bulk DE

Surface area has been related to pro-inflammatory responses [67, 68], and the surface area of processed DE may be a key factor controlling toxicity. Correlations between bulk surface area of processed samples (calcined and flux-calcined) and haemolysis or cell viability were observed (Fig. 10). In flux-calcined samples, up to 27 % of particles were >10 μm (Table 2), which may account for their low surface area (Table 1) and, accordingly, their low reactivity. However, the variable particle size distributions of unprocessed and calcined DE could not be correlated to their toxic potential (Additional file 1), and the correlation between surface area and reactivity could not be extended to unprocessed samples. This suggests that other parameters are responsible for their observed toxicity.

Fibres can cause frustrated phagocytosis, where macrophages struggle to fully engulf particles [69]. Fibre-like particles, up to 21 %, in calcined and unprocessed samples, were mainly in the form of diatom ‘rinds’ or small fragments of diatoms. The lower abundance in flux-calcined samples is likely due to sintering of fragments into large agglomerates. Previously, DE containing fibre-like particles (aspect ratio >3) was shown to be more potent in vitro than ‘non-fibrous’ DE [70], suggesting that particle morphology could play a role in the observed cytotoxicity. Particle morphology is important in phagocytosis [71] and, here, frustrated phagocytosis was observed and some fibre-like particles were not phagocytised. This was most evident for calcined and unprocessed samples (which had the highest % of fibre-like particles), where TNF-α production and LDH release, potential indicators of frustrated phagocytosis [72], were also elevated above flux-calcined samples. Frustrated phagocytosis was also observed as a number of cells attempted to engulf larger, disc-shaped diatoms. This was observed after treatment of cells with all of the fine fraction samples, suggesting that large, disc-shaped particles may play a role in DE toxicity, as platelets have been recently identified as a novel respiratory hazard [73], and DE particles with a diameter >7.5 μm have previously been suggested to determine DE toxicity [24].

## Conclusions

This study, the first to systematically characterise the physicochemical properties and potential toxicity of a range of globally sourced DE samples, shows that the toxic potential of DE varies by processing technique and source. Flux-calcined samples were unreactive, whereas unprocessed and calcined DE had variable reactivity.

No correlation was observed between crystalline silica content and DE’s potential toxicity, despite previously being implicated in epidemiological studies of DE exposure. The dearth of crystalline silica at the particle surface, due to its crystallisation within an amorphous matrix, its presence in a heterogeneous dust, and impurities within the crystalline silica likely reduce the potential reactivity of these crystalline silica-bearing particles.

It is likely that a number of physicochemical properties play a role in DE toxicity. Calcium-rich phases may be important in the toxicity of some unprocessed samples, and iron or amorphous phases may be involved in calcined DE toxicity. Surface area, especially, was correlated to calcined and flux-calcined DE reactivity here, and the importance of surface reactivity and the unique particle morphologies of DE merits further investigation.

Although no single physicochemical property of DE considered here could be linked to its potential toxicity, a clear outcome of this study is that the crystalline silica content, alone, should not be used to determine the DE hazard, nor should it be assumed that it is the cause of disease observed in epidemiological or clinical studies without further investigation.

## Additional file

Additional file 1:
**This document includes additional data including LDH and cytokine release data and correlations between and bulk Fe content and in vitro toxicity, and particle size and in vitro toxicity, which are all referred to in the main manuscript.**

## Abbreviations

DE: Diatomaceous earth
IMA: Industrial Minerals Association
XRD-PSD: X-ray diffraction position sensitive detection
XRF: X-ray fluorescence
SEM: Scanning electron microscopy
EDS: Energy dispersive X-ray spectroscopy
BET: Brunauer-Emmett-Teller
LDH: Lactate dehydrogenase
KC: Keratinocyte chemoattractant
IL-1β: Interleukin 1β
TNF-α: Tumour necrosis factor alpha
IL-10: Interleukin 10
