# Supplementary material for: The global variability of diatomaceous earth toxicity: a physicochemical and in vitro investigation
Source: J Occup Med Toxicol. 2015 Jul 10;10:23. doi: 10.1186/s12995-015-0064-7 (PMC4509483; doi:10.1186/s12995-015-0064-7)
Supplement: Additional file 1: — This document includes additional data including LDH and cytokine release data and correlations between and bulk Fe content and in vitro toxicity, and particle size and in vitro toxicity, which are all referred to in the main manuscript. [file 12995_2015_64_MOESM1_ESM.pdf]

The global variability of diatomaceous earth toxicity: a physicochemical and *in vitro* investigation

Nattrass, C., Horwell, C.J., Damby, D.E., Kermanizadeh, A., Brown, D.M., Stone, V.

SUPPLEMENTARY MATERIAL

The toxicity of soluble components of DE

To determine if soluble components of DE were haemolytic, two highly haemolytic DE powders (DE_05, DE_11) were leached in saline for one hour at the top treatment concentration (1000 µg/ml) by shaking gently, the suspension was then centrifuged at 5000 rcf for 15 minutes and filtered through a 0.2 µm Nalgene syringe filter, and the collected leachates were used in the haemolysis assay. All samples used in the cytotoxicity assays were also leached in complete medium for 24 hours at 63 µg/ml (in the absence of cells), centrifuged at 5000 rcf for 10 minutes and the collected leachate used to treat the cells, and their toxicity assessed using the alamarBlue® assay.

The leachates were not haemolytic, nor cytotoxic in the alamarBlue® assay and, therefore, soluble components of the DE were not the cause of the observed haemolytic and cytotoxic responses, which must be particle dependent.

Results

Lactate Dehydrogenase release

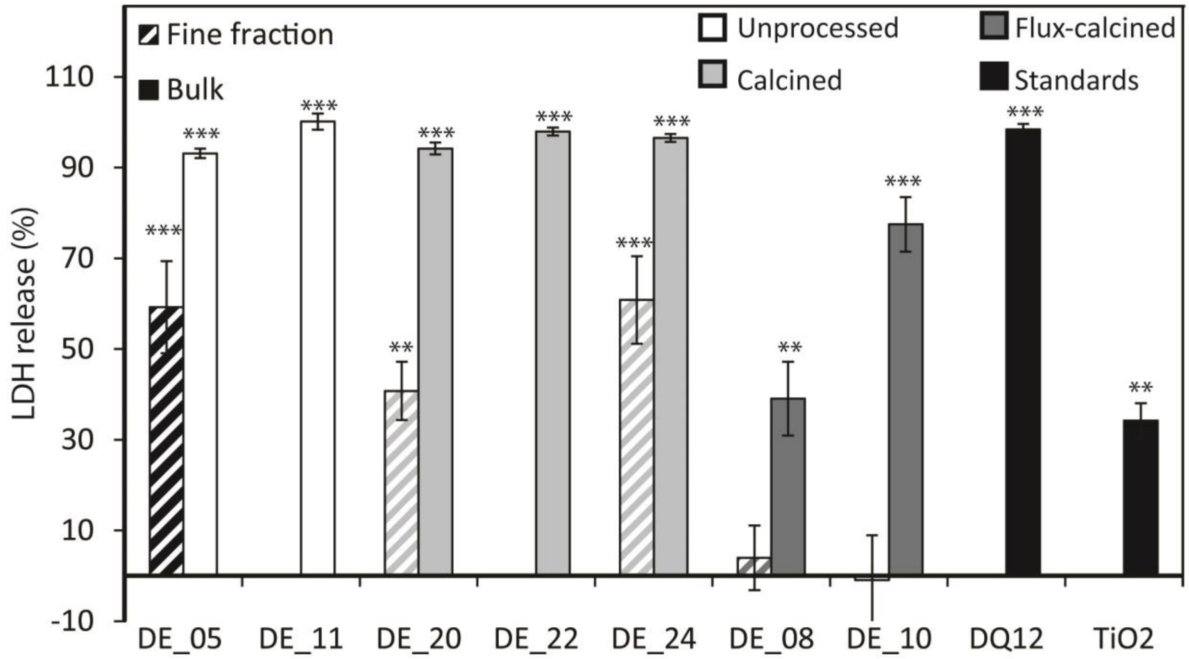

Figure S1: Lactate dehydrogenase release from J774 macrophages treated with 500 µg/ml of the fine fraction (hashed) and bulk (solid) samples of unprocessed (white), calcined (light grey), and flux-calcined (dark grey) DE for 24 h (n=4). Fine fractions were not analysed for DE_11 or DE_22. Error bars represent standard error (n=4), *** p = <0.001 difference from untreated control.

Cytokine production

Table S1: Cytokine release from J774 macrophages exposed to the separated fine fractions of DE for 24h. None of these cytokines were produced in concentrations significantly greater than the negative control.

**Sample**	**Cytokine release (pg/ml)**		
Concentration (µg/ml)	KC	IL-1β	IL_10
**DE_05**			
125	0.28	1.33	0.41
63	0.22	2.69	0.00
31	0.21	0.20	0.00
8	0.40	0.18	0.04
**DE_20**			
125	0.88	0.47	0.00
63	0.22	7.54	0.64
31	0.21	9.55	0.00
8	1.08	7.82	3.79
**DE_24**			
125	0.26	4.96	0.21
63	0.22	20.17	0.00
31	0.25	40.87	0.00
8	0.42	25.30	0.00
**DE_08**			
125	0.42	2.58	0.00
63			
31			
8			
**DE_10**			
125	0.75	16.56	1.90
63			
31			
8			
**DQ12**			
125	0.32	2.97	0.00
63	0.50	13.68	0.00
31	0.43	1.49	0.00
8			
**Untreated control**			
	0.27	13.65	0.07

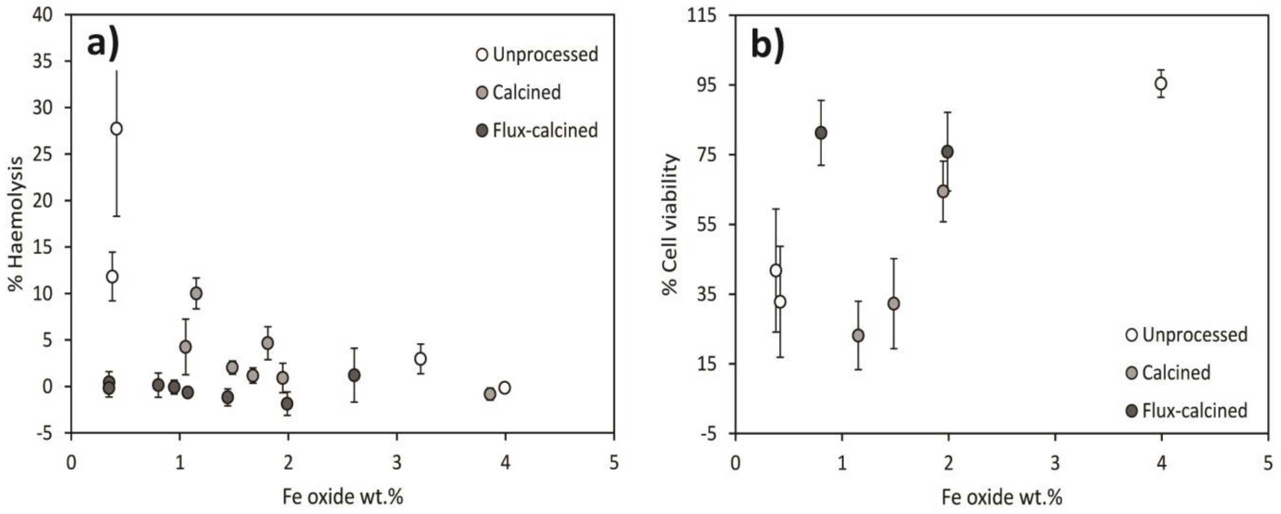

Figure S2: Correlation between bulk iron oxide content and **a)** haemolysis post-exposure to 1 mg/ml bulk DE, and **b)** cell viability post-exposure to 500 µg/ml bulk DE.

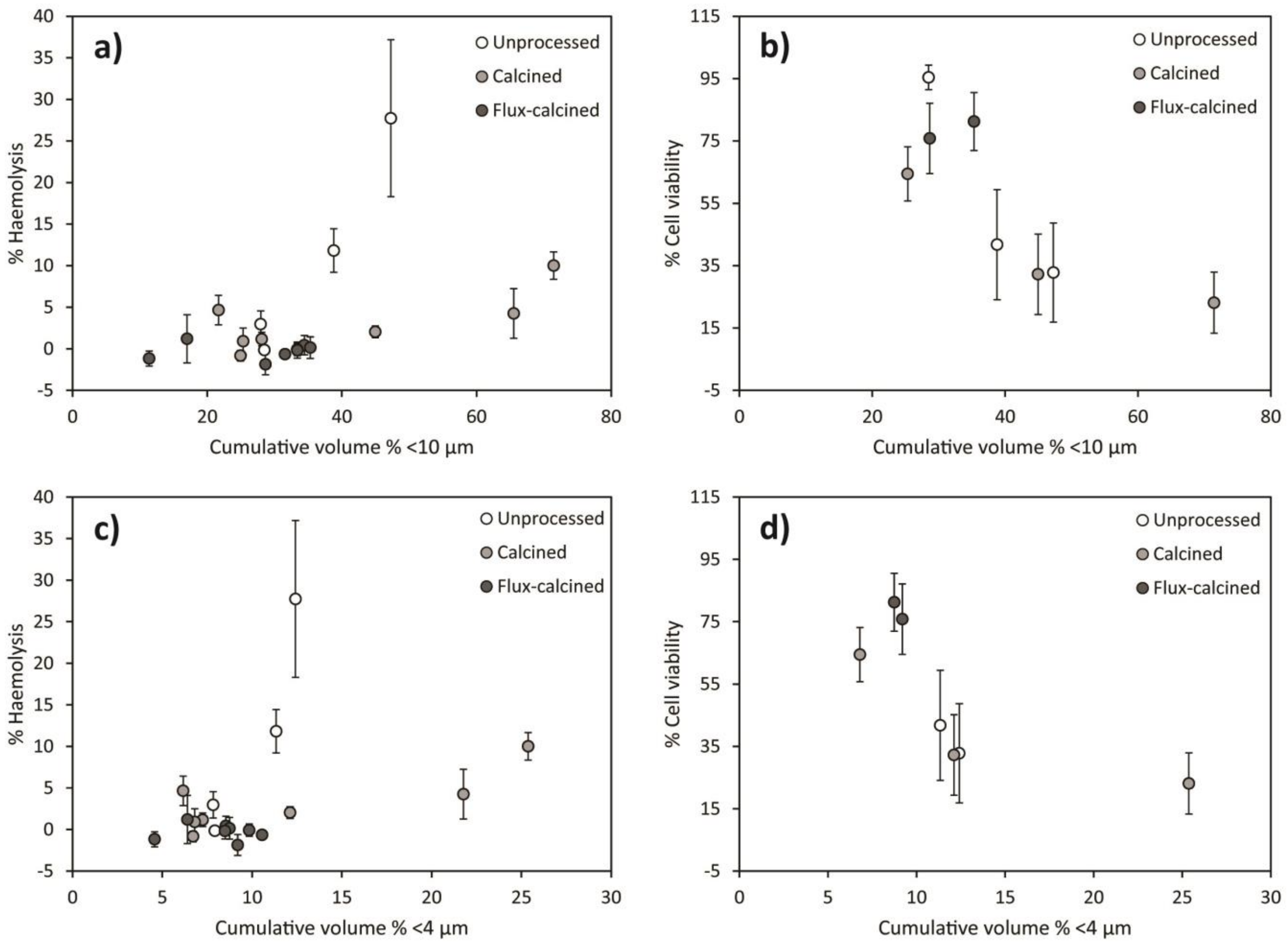

Figure S3: Correlation between cumulative volume % particle size <10 µm and **a)** haemolysis post-exposure to 1 mg/ml bulk DE, and **b)** cell viability post-exposure to 500 µg/ml bulk DE, and between cumulative volume % <4 µm **c)** haemolysis, and **b)** cell viability.
